# SARS-CoV-2 Spike Protein Activates Human Lung Macrophages

**DOI:** 10.3390/ijms24033036

**Published:** 2023-02-03

**Authors:** Francesco Palestra, Remo Poto, Renato Ciardi, Giorgia Opromolla, Agnese Secondo, Valentina Tedeschi, Anne Lise Ferrara, Rosa Maria Di Crescenzo, Maria Rosaria Galdiero, Leonardo Cristinziano, Luca Modestino, Gianni Marone, Alfonso Fiorelli, Gilda Varricchi, Stefania Loffredo

**Affiliations:** 1 Department of Translational Medical Sciences, University of Naples Federico II, 80131 Naples, Italy; 2 World Allergy Organization (WAO) Center of Excellence (CoE), 80131 Naples, Italy; 3 Translational Medical and Surgical Science, University of Campania Luigi Vanvitelli, 80131 Naples, Italy; 4 Department of Neuroscience, Reproductive and Odontostomatological Sciences, University of Naples Federico II, 80131 Naples, Italy; 5 Institute of Experimental Endocrinology and Oncology (IEOS), National Research Council, 80131 Naples, Italy; 6 Center for Basic and Clinical Immunology Research (CISI), University of Naples Federico II, 80131 Naples, Italy

**Keywords:** COVID-19, spike protein, ACE2, TMPRSS2, cytokines, macrophages

## Abstract

COVID-19 is a viral disease caused by SARS-CoV-2. This disease is characterized primarily, but not exclusively, by respiratory tract inflammation. SARS-CoV-2 infection relies on the binding of spike protein to ACE2 on the host cells. The virus uses the protease TMPRSS2 as an entry activator. Human lung macrophages (HLMs) are the most abundant immune cells in the lung and fulfill a variety of specialized functions mediated by the production of cytokines and chemokines. The aim of this project was to investigate the effects of spike protein on HLM activation and the expression of ACE2 and TMPRSS2 in HLMs. Spike protein induced CXCL8, IL-6, TNF-α, and IL-1β release from HLMs; promoted efficient phagocytosis; and induced dysfunction of intracellular Ca^2+^ concentration by increasing lysosomal Ca^2+^ content in HLMs. Microscopy experiments revealed that HLM tracking was affected by spike protein activation. Finally, HLMs constitutively expressed mRNAs for ACE2 and TMPRSS2. In conclusion, during SARS-CoV-2 infection, macrophages seem to play a key role in lung injury, resulting in immunological dysfunction and respiratory disease.

## 1. Introduction

SARS-CoV-2 is a single-stranded RNA virus responsible for the COVID-19 pandemic outbreak [1]. This disease is characterized primarily, but not exclusively, by respiratory tract inflammation. In most subjects, the infection leads to mild disease with symptoms including headache, fever, and cough. In the remaining cases, patients develop interstitial pneumonia requiring intensive care and oxygen support [1]. COVID-19 symptoms appear to be positively related to increased plasma levels of pro-inflammatory mediators, such as IL-1β, IL-1Ra, IL-4, IL-6, CXCL8, IL-10, IFN γ, and TNF-α [2,3].

SARS-CoV-2 virion is characterized by nucleocapsid, membrane, envelope, and spike protein [4]. The latter is assembled as a trimer, giving the appearance of a crown, and each monomer is formed by two subunits: S1 and S2 [4]. Spike protein plays a key role in the infection process enabling virus internalization by interacting with two receptors, angiotensin-converting enzyme 2 (ACE2) and transmembrane (TM) serine protease 2 (TMPRSS2) [4]. SARS-CoV-2 binds to the ACE2 receptor and fuses with the cell membrane [5]. In order to fuse with the cell membrane, spike protein must be cleaved into two domains (S1 and S2) by TMPRSS2, a type 2 TM serine protease located on the host cell membrane [6]. ACE2 and TMPRSS2 are expressed in different organs, including the lung and the heart [7,8]. This explains why the lung and, to some extent, the heart [9,10,11,12,13] are particularly vulnerable to SARS-CoV-2.

Macrophages are the most abundant immune cell type in lung tissue and play a key role in inflammatory and phagocytic processes through the release of cytokines (e.g., IL-6, IL-1β TNF-α, IL-12), chemokines (e.g., CXCL8), growth factors (e.g., VEGF, angiopoietins), and enzymes [14,15,16,17]. Previous studies have demonstrated that the lungs of individuals with COVID-19 showed dense infiltration of aberrantly activated tissue-resident and alveolar macrophages [18,19]. In particular, activated macrophages were abundant in the lungs of patients with severe COVID-19 [19,20]. Moreover, lung macrophages express genes associated with profibrotic functions in patients with severe COVID-19 [21].

There are no data on the expression of ACE2 and TMPRSS2, or the effects of the spike protein on human lung macrophages (HLMs). In this study, we have investigated the effects of spike protein on the release and expression of several cytokines and chemokines by HLMs and the expression of ACE2 and TMPRSS2 in these cells.

## 2. Results

### 2.1. Effects of Spike Protein on Cytokine and Chemokine Release from HLMs

**Figure 1:**
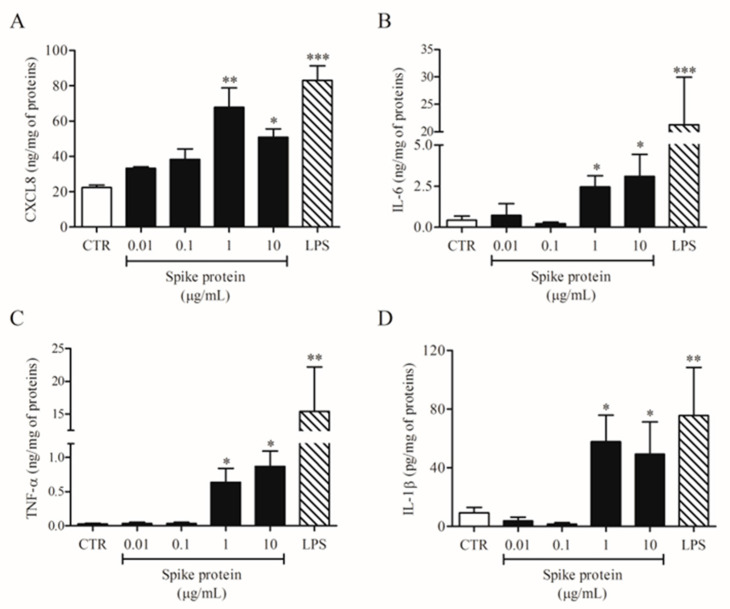
Effects of spike protein on cytokine and chemokine release from human lung macrophages (HLMs). HLMs (1 × 10^6^ cells/well) were incubated (18 h, 37 °C) with complete medium (CTR), or increasing concentrations of spike protein (0.01–10 μg/mL), or LPS (1 μg/mL). CXCL8 (**A**), IL-6 (**B**), TNF-α (**C**), and IL-1β (**D**) proteins in supernatants were evaluated by ELISA. Data are the mean ± SD of 8 experiments obtained from different donors. * *p* < 0.05; ** *p* < 0.01; *** *p* < 0.001 vs. CTR.

In a series of eight different experiments, we evaluated the effects of increasing concentrations of spike protein (0.01–10 µg/mL) on HLM activation. Spike protein activated HLMs inducing CXCL8 (Figure 1A), IL-6 (Figure 1B), TNF-α (Figure 1C), and IL-1β (Figure 1D) release. The spike protein effect was significant at 1 and 10 µg/mL. Conversely, spike protein had no effect on VEGF-A, ANGPT1, ANGPT2, and TGF-β release from HLMs (Supplementary Figure S1). In these experiments, we used LPS, the main component in the cell wall of Gram-negative bacteria, as a positive control [22]. We found that LPS is a potent stimulus for the release of all tested mediators except for TGF-β (Figure 1 and Supplementary Figure S1). The percentage of viable HLMs measured by MTT assay 18 h after spike protein treatment did not differ from that of untreated cells.

**Figure 2:**
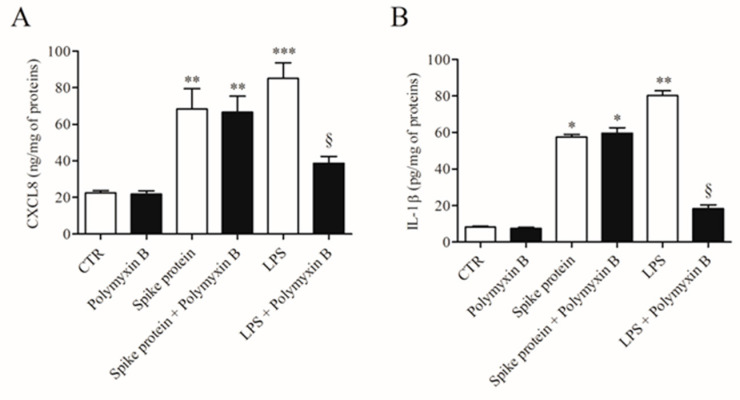
Effect of polymixin B on spike protein and LPS-induced release of CXCL8 and IL-1β from HLMs. HLMs were incubated (37 °C, 5% CO_2_, 16 h) with medium alone (CTR), spike protein (1 µg/mL), or LPS (1 µg/mL) either in the absence or in the presence of polymyxin B (50 µg/mL). Data are the mean ± SD of 3 experiments obtained from different donors. CXCL8 (**A**) and IL-1β (**B**) proteins in supernatants were evaluated by ELISA. * *p* < 0.05; ** *p* < 0.01; *** *p* < 0.001 vs. respective untreated (CTR) § *p* < 0.05 vs. LPS alone.

To exclude the possibility that the activating effects of spike protein could be due to minor LPS contamination, HLMs were stimulated with spike protein or in the presence of polymyxin B (50 µg/mL), a potent inactivator of LPS [23]. Figure 2 shows that polymyxin B did not modify the spontaneous release of both CXCL8 and IL-1β. Incubation of spike protein with polymyxin B did not modify the activating property of spike protein on the release of both CXCL8 and IL-1β from HLMs. As a control, polymyxin B significantly reduced the release of cytokines/chemokines induced by LPS.

**Figure 3:**
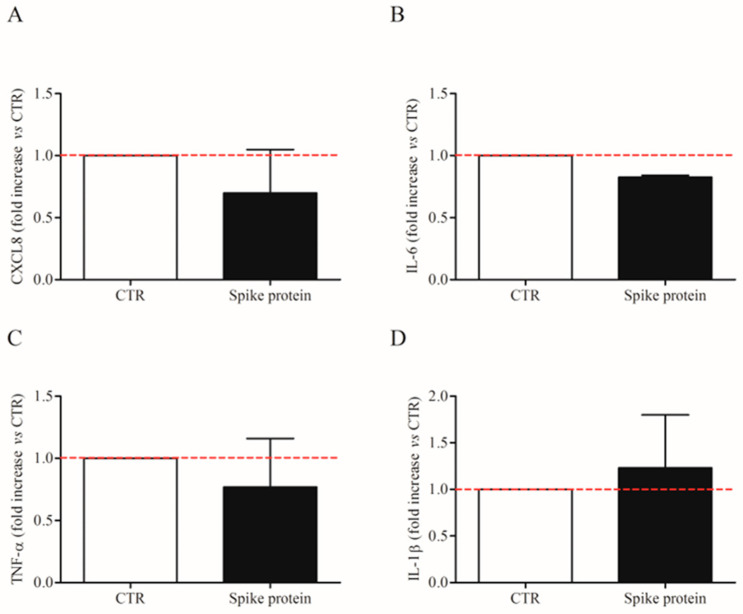
Effects of spike protein genes expression in human lung macrophages (HLMs). HLMs (3 × 10^6^ cells/well) were incubated (6 h, 37 °C) with complete media (CTR) or spike protein (1 μg/mL). At the end of incubation, HLMs were lysed and RNA was extracted. mRNA expression for CXCL8 (**A**), IL-6 (**B**), TNF-α (**C**), and IL-1β (**D**) was evaluated by quantitative RT-PCR. The red dotted line for each panel represents the control values. Data are the mean ± SD of 4 experiments obtained from different donors.

We also evaluated the effects of the lowest effective concentration of spike protein (1 µg/Ml; 7.4 nM) on *CXCL-8*, *IL-6*, *TNF-α*, and *IL1-β* mRNA expression in HLMs by real-time quantitative PCR. The stimulation of HLMs with spike protein for 6 h did not induce the mRNA expression for the tested mediators (Figure 3A–D). These results indicate that spike protein induced cytokine release from HLMs but not their de novo synthesis.

Previous studies showed that SARS-CoV-2 can activate monocytes and macrophages through spike interaction with TLR4 and TLR2 on cell lines and mouse models [24,25]. To evaluate whether TLR2 was involved in HLM activation induced by spike protein, we preincubated HLMs with anti-TLR2 and then stimulated them with spike protein. Anti-TLR2 had no effect on CXCL8, IL-6, and TNF-α release induced by spike protein (Supplementary Figure S2), suggesting that TLR2 is not required for cytokine and chemokine release from HMLs.

### 2.2. Effects of Spike Protein on Ca^2+^ Homeostasis

**Figure 4:**
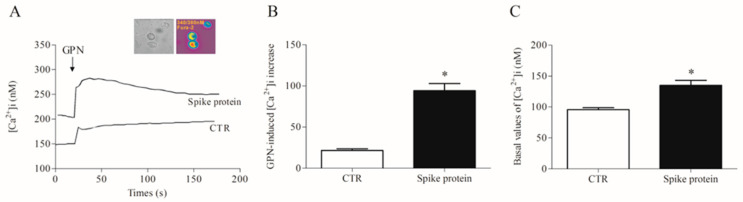
Effects of spike protein on cytosolic and lysosomal Ca^2+^ levels in human lung macrophages (HLMs). (**A**) Representative traces showing the effect of GPN (300 µM) on intracellular Ca^2+^ concentration [Ca^2+^]_i_) in HLMs stimulated (18 h, 37 °C) with RPMI alone (CTR) or spike protein (1 μg/mL). [Ca^2+^]_i_ was determined by a single-cell computer-assisted video imaging system. The insert depicts representative bright-field and ratiometric images of the HLMs loaded with Fura-2. (**B**) Bar graph depicting the quantification of [Ca^2+^]_i_ increase after GPN perfusion (300 µM). Each bar represents the mean ± SE (n = 15 cells for each treatment studied in three different experimental sessions). * *p* < 0.05 vs. CTR. (**C**) Bar graph depicting the basal values of [Ca^2+^]_i_ in CTR- and spike protein-activated HLMs. Each bar represents the mean ± SE (n = 25 cells for each treatment studied in three different experimental sessions). * *p* < 0.05 vs. CTR.

Most of the macrophage activation mechanisms are Ca^2+^-dependent [26,27]. Furthermore, the lysosomal two-pore Ca^2+^ channel has been proposed as a putative target to inhibit SARS-CoV-2 infection [28,29]. Therefore, we evaluated the effects of recombinant spike protein on lysosomal and cytosolic calcium concentration [Ca^2+^]_i_ in HLMs. After incubation with the spike protein (1 µg/mL/1 h), we detected a significantly increased lysosomal Ca^2+^ content than in control cells, as measured by the glycyl-L-phenylalanine 2-naphthylamide (GPN) addition (Figure 4). Being a cathepsin C substrate, GPN evokes a lysosomal content leak of Ca^2+^ that can be measured in the cytosol, thus providing an indirect measure of lysosomal Ca^2+^ content [30].

### 2.3. Effects of Spike Protein on Morphology and Kinetic Properties of HLMs

**Figure 5:**
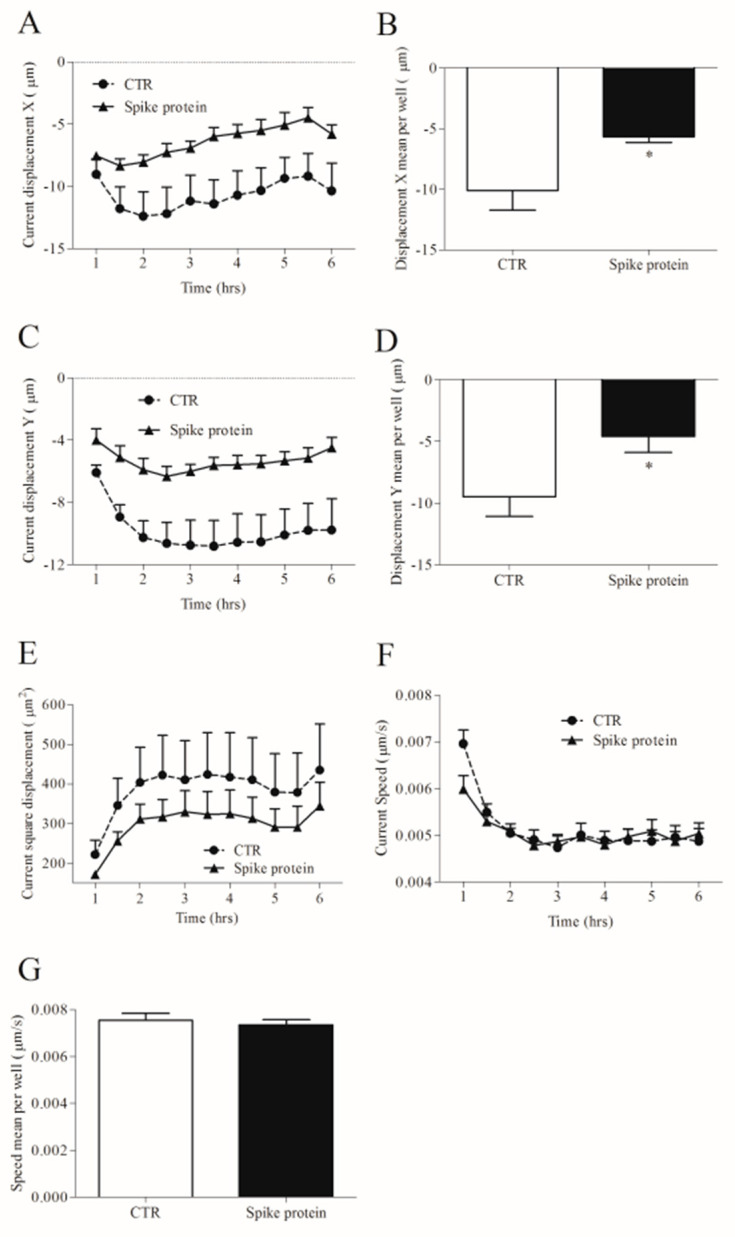
Effects of spike protein on kinetic properties of human lung macrophages (HLMs). HLMs (150 × 10^3^ cells/well) were incubated (6 h, 37 °C) with RPMI alone (CTR) or spike protein (1 μg/mL). The incubation was carried out with time-lapse and high-content microscopy Operetta High-Content Imaging System (PerkinElmer) to investigate the tracking characteristics as current displacement X (**A**), displacement X mean per well (**B**), current displacement Y (**C**), displacement Y mean per well (**D**), current square displacement (**E**), current speed (**F**) and speed mean per well (**G**). Data are the mean ± SD of 5 experiments obtained from different donors. * *p* < 0.05.

Different stimuli can change cell morphology as well as their movement [31]. We investigated HLM tracking and morphology with an Operetta High-Content Imaging System (PerkinElmer). To this end, HLMs were incubated with spike protein (1 µg/mL) or complete medium alone for 6 h at 37 °C and 5% of CO_2_. Spike protein did not influence HLM morphology compared to untreated HLMs (Supplementary Figure S3A–D). Regarding HLM tracking, when the starting point was set to 0 on the *X*-axis and 0 on the *Y*-axis, we found that spike protein induced changes in cell displacement. These were measured as current displacement X (Figure 5A), displacement X mean per well (Figure 5B), current displacement Y (Figure 5C), and displacement Y mean per well (Figure 5D). In particular, spike protein-stimulated HLMs were closer to the point of origin (Figure 5A–D), and accumulated in a smaller displacement area (Figure 5E) compared to unstimulated cells. Conversely, spike protein had no effect on the speed of HLMs (Figure 5E,F).

### 2.4. Effects of Spike Protein on Phagocytosis

**Figure 6:**
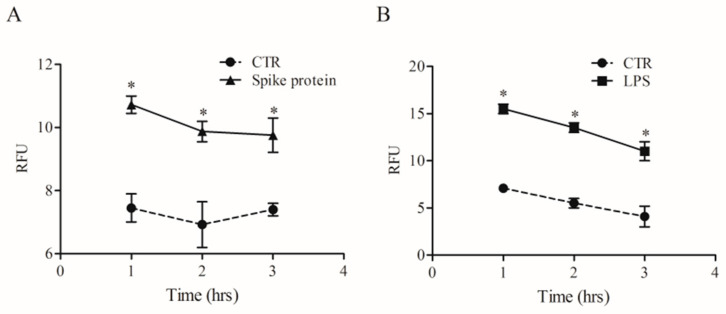
Effects of spike protein on human lung macrophages (HLMs) phagocytosis. HLMs (1 × 10^6^ cells/well) were incubated (37 °C, 5% CO_2_) with RPMI alone (CTR), and with spike protein (1 µg/mL) (**A**), or LPS (1 µg/mL) (**B**) one day before phagocytosis assay. The day after, the cell culture medium was removed and pHrodo™ Green *E. coli* BioParticles™ Conjugate suspension was added to the wells. The plate was placed in an EnSpire Multimode Plate Reader (PerkinElmer). The data were expressed as Relative Fluorescence Units (RFU) measured up to 3 h with a 1 h span at an excitation wavelength of 509 nm, and emission at 533 nm. Data are the mean ± SD of 5 experiments obtained from different donors. * *p* < 0.05 when compared to the corresponding value of CTR.

Phagocytosis is a fundamental mechanism of innate immunity that plays a key role in the elimination of microbial agents [32]. We evaluated whether spike protein can induce phagocytosis by HLMs. To this end, we used high-content imaging to measure the uptake of pHrodo™ green labeled *E. coli* particles over time in the same well. HLMs were seeded in imaging-compatible plates and then activated (18 h) with medium alone, spike protein (0.1 µg/mL), or LPS (1 µg/mL). At the end of incubation, pHrodo™ green labeled *E. coli* particles were added to HLMs, and phagocytosis of *E. coli* particles was monitored for 4 h. HLMs activated with spike protein showed higher uptake of the *E. coli* bacterial particles compared to the control (Figure 6A). As previously reported [33], LPS induced a remarkable and fast uptake of *E. coli* particles (Figure 6B) compared to untreated HLMs.

### 2.5. Expression of ACE2 and TMPRSS2 on HLMs

**Figure 7:**
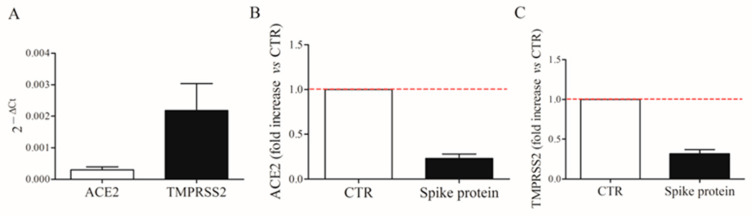
Constitutive expression of ACE2 and TMPRSS2 mRNAs in human lung macrophages (HLMs). HLMs (5 × 10^6^ cells) were lysed and RNA was extracted. ACE2 and TMPRSS2 mRNAs were determined by quantitative RT-PCR (**A**). HLMs (5 × 10^6^ cells/well) were incubated (6 h, 37 °C) with complete media (CTR), or spike protein (1 μg/mL). At the end of incubation, HLMs were lysed and RNA was extracted. mRNAs expression for ACE2 (**B**) and TMPRSS2 (**C**) was evaluated by quantitative RT-PCR. The red dotted line for each panel represents the control values. Data are the mean ± SD of 4 experiments obtained from different donors.

Spike protein allows the virus internalization by interacting with ACE2 receptors and TMPRSS2 on the cell surface [4]. We first evaluated the constitutive mRNA expression of *ACE2* and *TMPRSS2* in fresh HLMs by real-time quantitative PCR. HLMs constitutively express ACE2 and TMPRSS2 mRNAs (Figure 7A). TMPRSS2 is most abundantly expressed. Then, we investigated the effect of spike protein (1 µg/mL) on *ACE2* and *TMPRSS2* mRNA expression in HLMs. Figure 7 shows that spike protein did not affect mRNA expression for *ACE2* (panel B) and *TMPRSS2* (panel C).

## 3. Discussion

In the present study, we have demonstrated that incubation of HLMs with spike protein induces the release of pro-inflammatory mediators (i.e., CXCL8, TNF-α, IL-6, IL-1β), and an increase of intracellular Ca^2+^ concentration [Ca^2+^]_i_ and phagocytic functions. Moreover, spike protein affects macrophage tracking but not morphology. ACE2 and TMPRSS2, responsible for SARS-CoV-2 internalization, are constitutively expressed in primary macrophages purified from HLMs.

The lung is the primary site of SARS-CoV-2-induced immunopathology. The virus, thanks to spike protein, binds to ACE2 receptors and TMPRSS2, a cell surface protease that cleaves the spike protein thereby facilitating cell entry and infectivity [4]. Macrophages are the most abundant immune cells in the lung [34,35]. Previous studies have shown that mouse lung macrophages expressed ACE2 and TMPRSS family members [36]. Our study demonstrates for the first time that HLMs express mRNA for ACE2 and, to a greater extent, for TMPRSS2. These results suggested that HLMs are a target of the spike protein.

Macrophages are one of the main innate immune cells [35]. As part of innate immunity, lung epithelial cells are the first line to sense invading SARS-CoV-2 [37]. Cytokines, chemokines, and growth factors are then produced by the infected epithelium and resident immune cells, including macrophages, in the acute immune response [35,37]. A dysregulated macrophage activation has been observed in patients with severe COVID-19 and contributes to the development of acute respiratory distress syndrome (ARDS) [38,39]. Spike protein induces the release of IL-1β, IL-6, and TNF-α by MDMs, a model of monocyte-derived macrophages [40,41,42]. In this study, we demonstrate that primary HLMs exposed to spike protein release pro-inflammatory cytokines, including TNF-α, IL-1β, IL-6, and chemokines, such as CXCL8. The latter mediator can contribute to the recruitment of various inflammatory cells, such as neutrophils, monocytes, and natural killer cells to build a powerful innate immune defense system [43]. In our experimental conditions, spike protein induces the release of preformed mediators and has no effect on cytokine gene expression.

An aberrant innate immune response followed by altered adaptive immunity may cause pathogenic inflammation in the lungs of COVID-19 patients [20]. There is evidence that a critical disease is associated with a reduced response to IFN [44] and with the violent release of proinflammatory cytokines (i.e., TNF-α, IL-6) and chemokines (i.e., CXCL8) [2,45]. It is likely that the HLM activation by spike protein can trigger increased levels of proinflammatory cytokines in these COVID-19 patients. In addition, severe COVID-19 patients have increased circulating levels of angiogenic factors, such as VEGF-A, ANGPT1, ANGPT2 [46], and TGF-β [47]. Interestingly, although macrophages are a major source of these mediators [14,16,48], spike protein has no effect on their release. There is the possibility that other resident lung cells, such as mast cells, could be the source of angiogenic factors and TGF-β in COVID-19 patients [16].

Another interesting result is that spike protein modulated the intracellular Ca^2+^ concentration ([Ca^2+^]_i_) without producing HLM cytotoxicity. In particular, this recombinant protein triggered an accumulation of Ca^2+^ in lysosomes, probably as a result of its interaction with the acidic organelles. This subcellular interaction has been hypothesized by many research groups who identified lysosomal two-pore channels (TPCs) as a putative target of the virus [28,29]. Of note, it has been demonstrated that it is not the global Ca^2+^ signal, but local lysosomal Ca^2+^ nanodomains produced by the Ca^2+^ release through TPCs that drive macrophage phagocytosis [49]. Furthermore, while local Ca^2+^ nanodomains drive phagocytosis, the global Ca^2+^ signals may serve other roles, including mitochondrial ATP synthesis, changes in cytokine secretion, gene expression, etc. In this respect, our data show the ability of spike protein to evoke a [Ca^2+^]_i_ increase, an event possibly boosting the release of cytokines from HLMs. However, we cannot exclude the possibility that the [Ca^2+^]_i_ increase may also be a secondary event triggered by cytokine release.

There are three viral proteins on the surface of β-Coronaviruses, namely, spike protein envelope (E) protein and membrane (M) protein [50]. It was previously reported that purified E protein from SARS-CoV-2 can induce the release of several inflammatory cytokines (i.e., IL-1, IL-6, TNF-α, GM-CSF) and chemokines (i.e., CXCL10, MCP-1, CCL3) through the engagement of TLR2 on macrophages [51]. Moreover, SARS-CoV-2 can activate monocytes and macrophages through spike protein interaction with TLR4 and TLR2 on cell lines and mouse models [24,25]. Our results extend a previous observation showing that spike protein can induce the release of proinflammatory cytokines and chemokines from primary HLMs. Interestingly, our results show that this effect is, apparently not mediated by TLR2 because the antibody anti-TLR2 does not block the effect of spike protein on HLM activation. Our findings differ from previous results [24,25,51] indicating that TLR2, present on HLMs [52], is not involved in the release of cytokines from primary HLMs. Different experimental conditions could explain these apparent differences in results.

Collectively, these findings might explain the hyperactive cytokine release or cytokine storm associated with severe COVID-19. Identifying the sensors and ligands upstream of cytokine production in response to SARS-CoV-2 infection may provide critical information for clinical trials of drugs targeting cytokine storms.

Upon lung injury or infection, macrophages enhance phagocytic capacity [53]. Similarly, spike protein activates phagocytic processes and modifies physiological HLM movement. In particular, our results show that fewer spike protein activated-HLMs move from the start point compared to unstimulated cells, without altering the original speed and morphology.

## 4. Materials and Methods

### 4.1. Reagents

The following reagents were purchased: L-glutamine, fetal bovine serum (FBS), LPS (from *Escherichia coli* serotype 026:B6), Percoll^®^, piperazine- N, N_-bis-2-ethanesulphonic acid (PIPES), phosphate buffer saline (PBS), Triton X-100, antibiotic–antimycotic solution (10,000 IU/mL penicillin, 10 mg/mL streptomycin and 25 µg/mL amphotericin B) (Lonza, Basel, CH), RPMI 1640 (Microgem, Naples, Italy). Target-specific primers for CXCL8, IL-6, TNFα, IL-1β, ACE2, TMPRSS2, and GAPDH were designed using the Beacon Designer 3.0 (Bio-Rad Laboratories, Milan, Italy) and produced and purified by Custom Primers (Life Technologies, Milan, Italy). DMSO (Merck Millipore, Burlington, MA, USA). Polyclonal antibody to human TLR2 (anti-TLR2) (InvivoGen, Rho, Italy).

### 4.2. Isolation and Purification of Human Lung Macrophages (HLMs)

Macrophages were purified from macroscopically normal lung tissue of patients (hepatitis C virus−, hepatitis B surface Ag−, HIV1−) undergoing lung resection. The study protocol was approved by the Ethics Committee of the University of Naples Federico II (Prot. 7/19), and informed consent was obtained from patients undergoing thoracic surgery. Lung tissue was minced finely with scissors, washed with PIPES buffer over Nitex cloth (120 µm pore size; Sefar Italia), and the dispersed cells recovered. The macrophage suspension was enriched (75–85%) by flotation over discontinuous Percoll^®^ density gradients. The cells were suspended (10^6^ cells/mL) in complete medium (RPMI 1640 supplemented with 5% FCS, 2 mM L-glutamine, 1% antibiotic–antimycotic solution, and 1% non-essential amino acids) and incubated in 24-well plates at 37 °C. After 18 h, the medium was removed, and the plates were gently washed with fresh medium. More than 98% of adherent cells were macrophages, as evaluated by flow-cytometric analysis as previously demonstrated [54].

### 4.3. SARS-CoV-2 Spike Glycoprotein Stimulation of HLMs

The SARS-CoV-2 (2019-nCoV) Spike S1 + S2 ECD-His recombinant protein (spike protein) was obtained from Sino Biological (40589-V08B1) (Bio-Rad Laboratories, Segrate MI, Italy), reconstituted and used as recommended. The spike protein was diluted in fresh RPMI 1640 medium supplemented with 5% FBS before each experiment. HLMs were cultured in 24-well plates (0.15–2 × 10^6^ cells/well) in complete medium and then stimulated with LPS (1 µg/mL), or increasing concentrations of SARS-CoV-2 protein (0.01–10 µg/mL). In selected experiments, HLMs were preincubated (20 min a 37 °C, 5% CO_2_) with anti-TLR2 (5 µg/mL) and then stimulated with LPS (1 µg/mL) or spike protein (1 µg/mL; 7.4 nM). At the end of incubations, supernatants were harvested, centrifuged (300× *g*, 4 °C, 5 min), and stored at −80 °C for subsequent determination of mediator release. Lysis of the remaining cells in the plates was carried out using 0.1% Triton X-100 for total protein quantification by a Bradford assay (Bio-Rad Laboratories, Segrate MI, Italy).

### 4.4. Cell Viability

After treatments, cell viability was evaluated as mitochondrial activity, determined by the MTT (3-(4,5-dimethylthiazol-2-yl)2,5-diphenyl tetrazolium bromide) assay, as reported previously [55]. HLMs were incubated with spike protein, 1% *v*/*v* Triton X-100, or medium alone for the time indicated. At the end of the incubation, supernatants were removed, and the cells were incubated (37 °C, 1 h) in 1 mL of MTT solution (0.5 mg/mL). The cells were washed with PBS, 0.5 mL of DMSO was added, and absorbance was read at 540 nm. Cell injury was expressed as a percentage of cultures treated with medium alone.

### 4.5. ELISA Assays

The release of soluble mediators in the supernatants of HLMs was measured in duplicate using commercially available ELISA kits for CXCL8, IL-6, TNF-α, angiopoietin 1 (ANGPT1), ANGPT2, vascular endothelial growth factor (VEGF)-A, TGF-β (R&D Systems, Minneapolis, MN, USA), and IL-1β (Thermo Fisher Scientific Wilmington, DE, USA). Since the number of HLMs can vary among wells and different experiments, the results obtained were normalized for the total protein content in each well, determined in the cell lysates (0.1% Triton X-100). Therefore, all cytokine values were expressed as ng/mg of total proteins.

### 4.6. RT-PCR

*ACE2*, *TMPRSS2*, *CXCL8*, *IL-6*, *TNF-α*, and *IL-1β* mRNA expressions were investigated. HLMs (2 × 10^6^ cells/well) were incubated with LPS (1 µg/mL) and spike protein (1 µg/mL). After stimulation, supernatants were removed and HLMs were lysed to evaluate mRNA expression. *ACE2* and *TMPRSS2* mRNA expression was also evaluated in fresh lysed cells. Total RNA was extracted by TRIzol^®^ reagent (Euroclone, Milan, Italy) following the manufacturer’s instructions. RNA quality and integrity were estimated by spectrophotometric analysis on a Nanodrop ND-1000 spectrophotometer (Thermo Fisher Scientific, Wilmington, DE, USA). Reverse transcription was performed using the High-Capacity cDNA Reverse Transcription Kit (Applied Biosystems, Foster City, CA, USA). Real-time RT-PCR was performed by means of Universal SYBR Green Supermix (Bio-Rad) on a CFX96 real-time detection system (Bio-Rad Laboratories, Segrate, MI, Italy). GAPDH was used as a housekeeping gene to normalize Ct (cycle threshold) values using the 2^−ΔCt^ formula. PCR efficiency and specificity were evaluated by analyzing amplification curves with serial dilutions of the template cDNA and their dissociation curves. Each cDNA sample was analyzed in triplicate and the corresponding no-RT mRNA sample was included as a negative control. The data were analyzed with iCycleriQ analysis software (Bio-Rad Laboratories, Segrate, MI, Italy), and the changes in *CXCL8*, *IL-6*, *TNF-α*, *IL-1β*, *ACE2*, and *TMPRSS2 mRNAs* were expressed as 2^−ΔCt^.

### 4.7. [Ca^2+^]_i_ Measurement in Single-Cell

HLMs cultured on poly-L-lysine–coated glass coverslips were loaded with 10 µM Fura-2 AM for 1 h at 37 °C in Krebs-Ringer saline solution (containing the following: 5.5 mM KCl, 160 mM NaCl, 1.2 mM MgCl_2_, 1.5 mM CaCl_2_, 10 mM glucose, and 10 mM HEPES-NaOH (pH 7.4). At the end of the loading period, [Ca^2+^]_i_ was monitored using single-cell computer-assisted video imaging [56] with a digital imaging system composed of a Zeiss Axiovert 200 microscope (Carl Zeiss, Jena, Germany) equipped with a FLUAR × 40 oil objective lens, MicroMax 512BFT cooled charge-coupled device camera (Princeton Instruments, Trenton, NJ, USA), LAMBDA 10–2 filter wheeler (Sutter Instruments, Novato, CA, USA), and MetaMorph/MetaFluor Imaging System software (Universal Imaging, West Chester, PA, USA). The coverslip containing cells was placed on a perfusion chamber (Medical Systems, Greenvale, NY, USA) and illuminated alternately at 340 and 380 nm by a Xenon lamp. The emitted light was passed through a 512-nm barrier filter. Fura-2 AM fluorescence intensity was measured every 3 s. A total of 15–20 individual cells were selected and monitored simultaneously from each coverslip. The results have been presented as cytosolic [Ca^2+^]. Lysosomal Ca^2+^ content was measured as the [Ca^2+^]_i_ increase induced by the addition of the lysosomotropic agent glycyl-L-phenylalanine 2-naphthylamide (GPN). GPN is able to probe lysosomal Ca^2+^ content by triggering a rapid [Ca^2+^]_i_ peak through lysosomal osmotic rupture [57]. Calibrations used the equation of Grynkiewicz et al., assuming that the K_D_ for Fura-2 AM was 224 nM [58]. [Ca^2+^]_i_ was measured in HLMs incubated with only medium and spike protein (1 µg/mL).

### 4.8. Time-Lapse and High-Content Microscopy

Microscopy experiments were conducted with the Operetta High-Content Imaging System (PerkinElmer, MA, USA), as previously described [59,60]. HLMs were cultured in Falcon^®^ 24-well Clear Flat Bottom plates. For time-lapse experiments, HLMs were stimulated with spike protein (1 µg/mL) and cultured for 6 h. Within this time window, digital phase contrast images of 15 fields/well were captured every 10 min via a 10× objective. To quantify cell morphological and tracking features, bright-field snapshots were taken at 6 fields/well. PhenoLOGIC (PerkinElmer) was employed for image segmentation and for calculating the single-cell morphological results by the dedicated STAR analysis sequence [59]. STAR morphology is an enhanced series of algorithms that provide a statistically powerful set of properties for analyzing phenotypes by characterizing cell morphology and the intensity distribution within regions. The STAR method can calculate symmetry properties, threshold compactness, axial properties, radial properties, and profile [59,60]. PhenoLOGIC (PerkinElmer) was also employed to analyze kinetic proprieties as current displacement X, current displacement Y, displacement X mean per well, displacement Y mean per well, current square displacement, displacement mean per well, and current speed.

### 4.9. Phagocytosis Assay

HLMs (1 × 10^5^ cells/well) were seeded into imaging-compatible plates (BD, Falcon) 1 day before the phagocytosis assay. Once adhered (37 °C, 5% CO_2_), HLMs were stimulated with spike protein (1 µg/mL). The day after, the cell culture medium was removed and pHrodo™ Green *E. coli* BioParticles™ Conjugate suspension was added to the wells. pHrodo™ Green conjugates are non-fluorescent outside the cell at neutral pH but fluoresce brightly green at acidic pH, such as in phagosomes. The plate was placed in an EnSpire Multimode Plate Reader (PerkinElmer). The data were expressed as relative fluorescence units (RFU) measured up to 3 h with a 1 h span at an excitation wavelength of 509 nm and emission at 533 nm.

### 4.10. Statistical Analysis

Statistical analysis was performed by using Prism 9 (GraphPad Software, San Diego, CA, USA). The data are expressed as mean values ± standard deviation (SD) of the indicated number of experiments. Data were compared by Student’s t-test or one-way analysis of variance (ANOVA) followed by Dunnett’s test (when comparison was made against a control) or Bonferroni’s test (when comparison was made between each pair of groups) by means of Analyse-it for Microsoft Excel, version 2.16 (Analyse-it Software, Ltd., Leeds, UK). A *p*-value ≤ 0.05 was considered statistically significant.

## 5. Conclusions

Our study has some limitations that should be pointed out. Although macrophages are the predominant immune cells in the human lung, single-cell transcriptomics has identified several subpopulations of HLMs [61,62]. Our experiments were performed using purified HLMs, as evaluated by flow-cytometric analysis [63]. Spike protein stimulates proinflammatory response (e.g., the release of cytokines and chemokines) and protective effects (e.g., phagocytosis). These divergent effects could be explained by the engagement of protein S on different subpopulations of HLMs. In addition, the in vitro experiments were carried out using primary macrophages obtained from patients undergoing thoracic surgery. We cannot exclude the possibility that the underlying disease may have influenced some of our results.

In conclusion, during the SARS-CoV-2 infection, macrophages seem to have advantageous and disadvantageous roles. Through phagocytic functions, they remove pathogens and cell debris, and through the release of proinflammatory mediators, contribute to lung injury resulting in respiratory dysfunction and disease.

## Data Availability

Data supporting the reported results are available upon request.

## Supplementary Materials

The following supporting information can be downloaded at: https://www.mdpi.com/article/10.3390/ijms24033036/s1.

## Abbreviations

ACE	angiotensin-converting enzyme
ANGPT	angiopoietin
Ct	cycle threshold
Ca^2+^	calcium concentrations
FCS	fetal calf serum
HLM	human lung macrophage
IL-	interleukin
LPS	lipopolysaccharide
MDM	monocyte-derived macrophage
PBS	phosphate buffer saline
PIPES	piperazine- N, N_-bis-2-ethanesulphonic acid
RT-qPCR	quantitative reverse transcriptase PCR
RFU	relative fluorescent unit
TNF	tumor necrosis factor
VEGF	vascular endothelial growth factor
