# Supplementary material for: SARS-CoV-2 Spike Protein Activates Human Lung Macrophages
Source: Int J Mol Sci. 2023 Feb 3;24(3):3036. doi: 10.3390/ijms24033036 (PMC9917796; doi:10.3390/ijms24033036)
Supplement: Supplementary file 1 [file ijms-24-03036-s001.zip › ijms-2194672-supplementary.pdf]

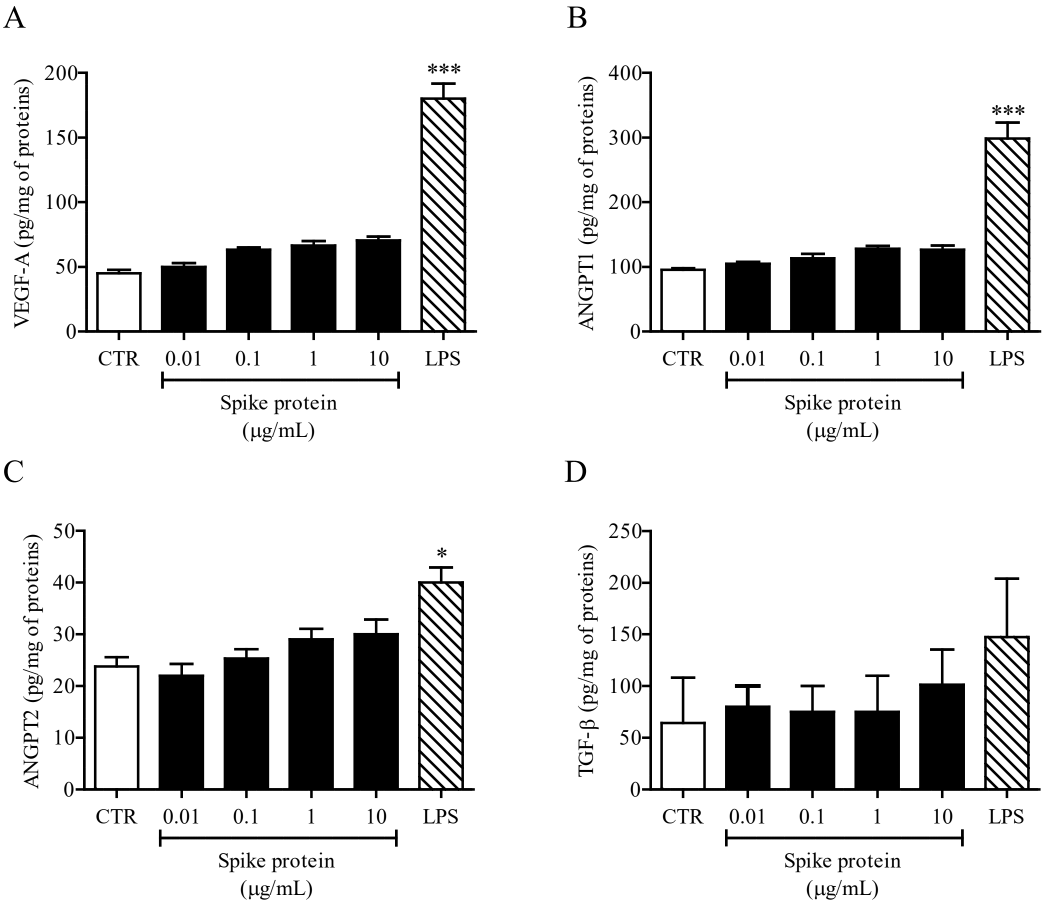

Figure S1. Effects of Spike protein on the release of vasoactive factors and TGF-β from human lung macrophages (HLMs). HLMs (1 x 10^6 cells/well) were incubated (18 hrs, 37°C) with complete media (CTR) or increasing concentrations of Spike protein (0.01-10 µg/mL) or LPS (1 µg/mL). VEGF-A (**A**), ANGPT1 (**B**), ANGPT2 (**C**) and TGF-β (**D**) proteins in supernatants were evaluated by ELISA. Data are the mean ± SD of 8 experiments obtained from different donors. *p<0.05; ***p<0.001 *vs* CTR.

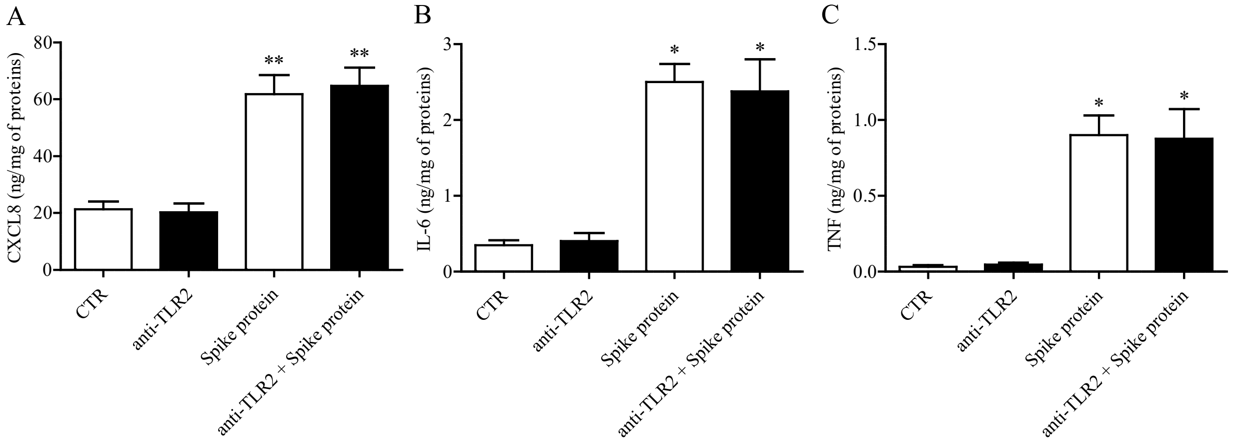

Figure S2. Effects of anti-TLR2 on cytokine and chemokine release from human lung macrophages (HLMs) induced by Spike protein. HLMs (1 x 10^6 cells/well) were preincubated (10 min, 37°C) with o without polyclonal antibody anti-TLR2 (5 µg/ml) and then stimulated (18 hrs, 37°C) with complete medium (CTR) or Spike protein (1 µg/mL). CXCL8 (**A**), IL-6 (**B**) and TNF-α (**C**) proteins in supernatants were evaluated by ELISA. Data are the mean ± SD of 4 experiments obtained from different donors. *p<0.05; **p<0.01 *vs* CTR and anti-TLR-2 alone.

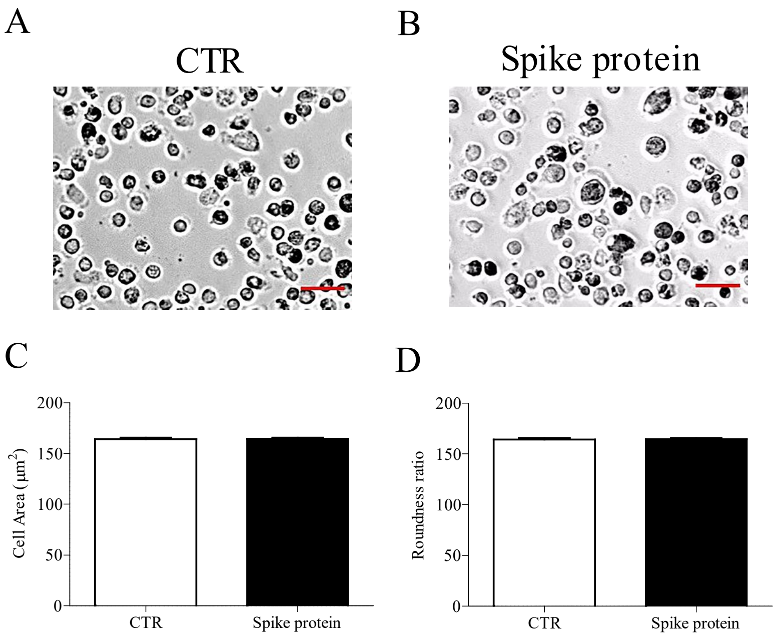

Figure S3. Effect of Spike protein on morphological changes in human lung macrophages (HLMS). HLMs (150 x 10^3 cells/well) were incubated (6 hrs, 37 °C) with RPMI alone (CTR) or Spike protein (1 µg/mL) and then were imaged by means of an Operetta high-content imaging system at 10× magnification. The images (**A** and **B**) were analyzed in the Harmony software with PhenoLOGIC (PerkinElmer) and a dedicated analysis sequence (morphological properties, method STAR) to evaluate cell area (**C**) and roundness (**D**). Scale Bar 50 µm (red line). Data are the mean ± SD of 5 experiments obtained from different donors.
